# Development of Creative Intelligence in Physical Education and Sports Science Students through Body Expression

**DOI:** 10.3390/ijerph18105406

**Published:** 2021-05-19

**Authors:** Andreea Vidaci, Lilyan Vega-Ramírez, Juan Manuel Cortell-Tormo

**Affiliations:** Department of General and Specific Didactics, Faculty of Education, University of Alicante, 03690 Alicante, Spain; av73@alu.ua.es (A.V.); jm.cortell@ua.es (J.M.C.-T.)

**Keywords:** creativity, sports, genders, university students, physical activity

## Abstract

Body expression can enhance movement creativity and at the same time promote the growth of creative intelligence in college age. The aim of this study was to analyze the influence of an intervention in body expression classes on the creative intelligence of university students. The 49 participants aged 19 to 38 years engaged in the body expression course for seven weeks, 3 h per week. The Creative Intelligence test (CREA) was applied as an evaluative method to obtain the initial data and after the intervention the test was reapplied. Pre- and post-test results were analyzed and compared by gender and type of sporting background (team or individual sports). The results indicate an overall improvement in creative intelligence with a significant difference between the two evaluations (*p* < 0.001). Women started with a higher score than men, and although an improvement in their final mean score could be observed, it was not significant. Men, on the other hand, had noted a significant increase (*p* < 0.001) of these values in the post-test. Regarding the type of sports, at the beginning of the study, both groups had similar results; however, in the final test, the team sports players obtained better scores. In conclusion, body expression, thanks to its content focused on artistic-creative development, has been shown to be useful in the general progression of creative intelligence in college age.

## 1. Introduction

Body expression, considered the oldest form of communication, was used by the human being to create, express and communicate feelings, emotions, and ideas in a deliberated and aesthetic manner [1]. A recent study affirms that body expression is a reliable method to understand the most natural meaning of a human’s expressiveness, and is it achieved by body awareness, self-knowledge and educational transformation [2]. It also represents the artistic, expressive element of physical activities that use innovative methodological approaches aimed at promoting autonomous learning and social skills [3]. Through body expression, students can acquire attitudes, concepts and procedures that can be transferred to their daily physical activity [4]. This might improve their quality of life by gaining body awareness, a better knowledge of body features and a proper control and use of emotions [5].

Body expression relies on a series of elements to create a pleasant and welcoming environment that facilitates communication and exposure to different moods [6]. Music is used as one of the main elements that eases the development of the activity during the classes. The musical experience that accompanies the movement not only provides basic melodic elements (rhythm, melody, and harmony), but also stimulates the interpretation of the emotion and the development of physical, cognitive, and social skills [7]. Other elements used within the subject are students’ features, the visual environment (lighting, space) [8], and together with social encounters and human connections [9], are vital in the evolution of creative thinking.

The creative process involved in the artistic and athletic practice of students has recently begun to be studied [10]. It has also been found that practitioners of body expression have obtained higher values in aspects of creativity such as fluency, flexibility, and expressiveness [11]. Body expression has been shown over the years as a necessary tool in the school curriculum to promote creativity [2].

The differences between genders in creativity are influenced by environmental factors: the differing opportunities available to men and women, and the kinds of experiences both genders are likely to have [12]. Recent studies had found that women show more interest and motivation to carry out artistic and language activities [13,14,15], for example, in dance and gymnastics. On the other hand, men prefer sports that are dominated by physical contact and strength [16].

The current need to provide new knowledge in different contexts of action turns creativity into a basic component for the integral development of the human personality [17]. Creativity represents a universal trait, and it refers directly to the existential and work patterns in daily life [18]. It also represents the capacity of the individual to generate new and ingenious ideas; ideas that in the future can be used to solve difficulties [19]. Therefore, creativity takes on greater importance during university training, when students prepare for the future tasks, they will have to perform as teachers/trainers, many situations requiring novel options and solutions. These arguments are in line with the acquisition of key competence for university students, established by the European Higher Education Area [17,20].

Creativity represents an important basis in decision-making situations during sports practice, especially in the competitive period, and it is often known as tactical creativity [21]. In high-performance sports, it is crucial to be able to surprise the opponent with the decision-making process in order to make it harder for them to predict what will happen next [22,23]. Higher tactical intelligence is correlated with higher levels of game and tactical creativity [22].

Depending on the type of sports, creative thinking gains more or less importance. Despite the artistic orientations of some individual sports, the need for creative development within team sports can be easily spotted [23,24]. It is not to be confused the athlete’s expert decision-making skills and their creative ability, which represent divergent and convergent thinking [21]. In order to increase creativity during sports training, the key was to balance of the organized sports and informal sports [25].

Assessing and quantifying creativity has represented a great challenge for specialists due to the absence of a correct “true-false” answer and due to the motivational factor required in creative performance [26,27]. The literature review showed that research has focused on the development of motor creativity through body expression [11,27,28,29]. The development of creative thinking and body expression in university students being less studied.

In this field, the main focus was on different aspects of development of motor creativity through body expression [11]. The relationship between neuroplasticity, cognitive and motor learning through dance program was also studied by several scientists with amazing outcomes including the development of grey matter [30,31,32]. Due to the medical analysis required to evaluate the brain structure, such as MRI or diffusion tensor imaging (DTI), the research in this field is limited. That is the reason why many body expressivity experts based their research on psychology evaluations instead [33]. Creativity is one of the main areas to have been analyzed using psychology tests. One study revealed that younger students are prone to improve their fluidity, flexibility, expressivity and originality in terms of the creative process, but the difference between the students involved in dance-related activities and non-practitioners is not significant [11]. Other research claimed that movement creativity could only be developed through social interaction and as a response to the gestures that one’s partner is making [34]. There were a few researches that analyzed the transfer between movement and general creativity. One of them studied the effects of body expressivity on verbal and graphic creativity but without significant outcomes [35]. The main reason of the insignificant results might be the instrument used in order to collect the data or even the evaluation method [36].

In order to solve the aforementioned difficulties, a cognitive measure has been developed to assess creativity based on the individual’s ability to generate questions related to images, called CREA. The interest is centered in the facility and disposition for the elaboration of new structures, no matter how simple and common they are. It has been observed that the versatility in the use of cognitive schemes is a field where the production of questions and creativity collide [36].

Considering all the above, the aim of this study was to analyze the influence of body expression on the development of creative intelligence and its relationship with gender and type of sports practice by students in Physical Activity and Sport Sciences. We hypothesized that creativity improves by participating on the classes of body expression.

## 2. Materials and Methods

### 2.1. Sample

The initial sample was made of 90 students from the second year of Physical Activity and Sport Sciences degree, during the academic year 2019–2020. After applying the exclusion criteria, the final sample of 49 participants aged 20.48 ± 3.62 years (women and men 21.56 ± 3.02 and 20.55 ± 3.89 years, respectively) has been obtained.

The exclusion criteria were:

- Attendance at classes less than 85%;
- Not completing the pre- or post-test.

Participants were informed that the collected data were used for research purposes. This way informed consent was obtained, following the personal data protection guidelines and the approval of the ethics committee of University of Alicante (UA-2020-11-21).

### 2.2. Instrument

The instrument used has been the CREA manual of creative intelligence [26]. This evaluation method uses as an easy way to measure creativity through a person’s ability to generate questions. As its authors say, the test represents a novel instrument for the assessment of creativity, which meets the standards of reliability and validity required for a psychological test [26]. Besides the accessible feature of the test, CREA stands out for its diversity and availability to be applied to different age groups: children, teenagers, or adults. As the authors suggested, for the age group (above 17 years old) of the selected sample, the sheets CREA A and CREA B were applied collectively by writing method. They had also offered a percentile scale for the Spanish sample in order to classify the scores in levels of creativity (Table 1).

The CREA test proved to be useful in the evaluation of cognitive flexibility by generating questions that represents a potential indicator of creativity due to the correlation between efficacy and originality [36].

Sheet A showed an image of an antique telephone and sheet B presented a utopist scene were the entire crowd had their ears removed. Any question from the origin of the object, materials, members, actions, use, relation between characters, image environment, etc., was valid.

### 2.3. Procedure

At the beginning of the course, the initial CREA test corresponding to sheets A and B were applied, with an anonymous socio-demographic questionnaire that has collected data on age, gender and the type of sport practiced (individual or team) since the participants were students of physical activity and sports sciences. Each participant had been assigned a code in order to associate the pre- and post-test results. The students were informed that the test consisted in asking the biggest number of questions possible over an image. The initial evaluation was carried out following the application guidelines of the CREA test. The test was made of two sheets (CREA A and CREA B) and the participants had 4 min to answer each sheet. The overall time of evaluation was 10 min. The test was applied again at the end of the intervention in order to know the evolution of the students’ creative capacity. The same application guidelines mentioned in the manual were followed as in the pretest.

The intervention was performed over 21 h of body expression lessons (Table 2) during 7 weeks/sessions. Thanks to the specific contents of body expression subject, that encompasses individuality, teamwork, interrelation and cooperation, the score in the creative assessment can been improved in a short time, a fact that is also corroborated by other studies [37].

### 2.4. Statistical Analysis

Descriptive statistics (mean and SD) were calculated for all dependent variables. Normality and homogeneity of all variables were tested with the Kolmogorov–Smirnov and the Levene test, respectively. A parametric Student’s *t*-test was used to establish pre-post differences in CREA, women, men, team and individual variables. In order to evaluate pairwise comparison, the effect size, d-Cohen test (ES) was used [38]. Interpretation of data was determined using three effect size categories (small 0.20, moderate 0.50 and large 0.80). For all statistical tests a probability level of *p* < 0.05 denoted statistical significance. Statistical analyses were conducted with the SPSS^®^ (v26.0; IBM^®^, Armonk, NY, USA).

## 3. Results

The sample was made of 49 participants (15 women and 34 men) and all of them participated in these activities for at least 3 h per week. From the 15 women, seven were team sports players (two basketball, two volleyball, two football and one handball) and eight practiced individual sports (three triathlon, two athletics, one fitness, one martial arts and one artistic gymnastics). In the group of 34 men, 23 were team players (19 football, three basketball and one volleyball player) and 11 were individual sports players (four cycling, two triathlon, two boxing, one athletics, one fitness and one tennis player).

Regarding the results obtained from the investigation of creativity advances through body expression, a general increase is observed in the second evaluation compared to the first, prior to the intervention. It started with a general mean direct score (DS) of CREA of 23.12 and a SD of 7.19 and after participation in the body expression activity, a significant improvement was noticed (*t* = −4.523; *p* < 0.001; ES = 0.4) until reaching a general mean of 26.20 and a SD 7.51.

Considering the scale of the Spanish sample offered by the test authors, we obtained the percentile score (PS). We observed that initially all the subjects were classified in the groups of low (1–25 PS) or medium (26–74 PS) and afterwards only two of them reached the high level of creativity (75–99 PS) (Table 3).

**Figure 1:**
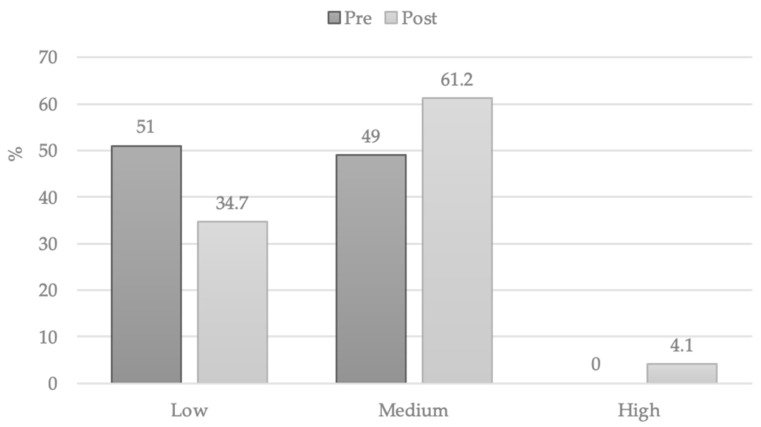
Changes in level score of CREA.

In Figure 1, it could be observed the migration within the levels of creativity from the initial to the final evaluation.

After segregating the data according to gender, the following observations were obtained:

- Women started with a higher score than men (24.20 mean and 7.804 SD), and although an improvement in their final average could be observed (26.47 mean and 7.990 SD), it was not significant (*t* = −2.041 *p* = 0.061 ES = 0.2).
- Men, on the other hand, in the pre-test achieved a lower mean (22.65 mean and 6.971 SD), but in the general mean of the post-test was noted a significant increase (*t* = −4.029 *p* < 0.001 ES = 0.6) of these values (26.09 mean and 7.412 SD).

In a comparison by levels of creativity (Table 4), a slight difference could be observed in the pre-test, where more than a half of the women were located in the medium level with 30.13 mean, while a smaller part of men got to the same level and obtained a mean of 28.56.

In the post-test, the percentages were surprisingly balanced between the two genders, a fact that denotes a higher increase in the levels of creativity in men, who even surpassed the score obtained by women in each level.

**Figure 2:**
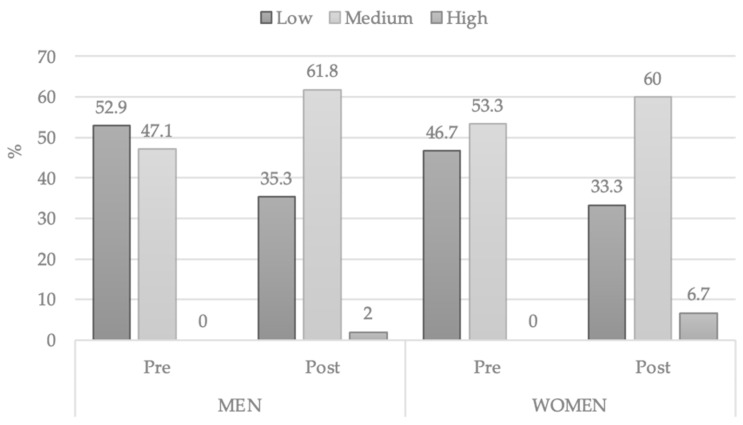
Changes by gender in level of CREA.

Figure 2 showed the percentages of men and women divided into the levels of creativity.

In the comparison by the type of sports affiliation, team or individual, some differences were also observed. The two groups started with a similar initial mean DS, but after intervention, only the participants of team sports showed significantly progress (*t* = −5033 *p* < 0.001) while individual sports players did not *(t* = −1.238 *p* = 0.231) (Table 5).

Both groups have been initially divided in two between the lowest level and the middle level of creativity, with a slight difference between the direct scores means. Team sports payers obtained a higher score in the low level while individual players got a better evaluation in the middle level of creative intelligence.

In the second evaluation, the students who were part of the team have progressed considerably, even reaching the highest level of creativity, unlike individual sports practitioners who had basically maintained the initial scores. It is noticeable that team players have progressed more after the intervention in the creativity intelligence evaluation levels. Table 6 presents the percentile scores obtained by the students, segregated by type of sports affiliation.

**Figure 3:**
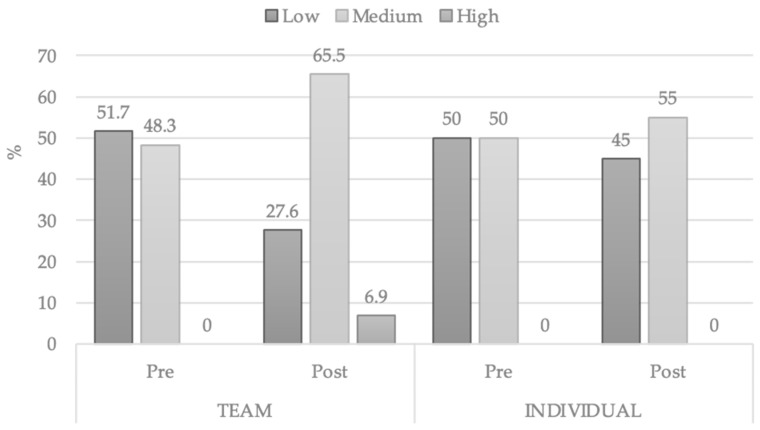
Changes in level of CREA by sport affiliation.

The percentages of team and individual sports players divided into levels of creativity are displayed in Figure 3.

## 4. Discussion

The aim of this study was to analyze the influence of body expression on the development of creative intelligence and its relationship with gender and type of sports practice by students of Physical Activity and Sport Sciences degree.

The overall results in the initial tests were slightly lower (mean 23.12) than other studies made with British college students in CREA test (26.61 mean) [39]. These results may be related to the low development of creativity within Spanish educational curricula [40]. In spite of only half of participants having achieved the medium level of general creativity, according to the test authors, they had a good capacity for adaptability, were very collaborative and had the potential to develop their creativity. The other half that was situated at the lowest level were defined by low conflict and were effective in structured or routine settings [26].

The post-test analysis showed a significant difference in the progress of the students’ creativity. The corporal, spatial and temporal exploration carried out for seven weeks aided the creative work. A small percentage advanced to the highest level but the vast majority were concentrated on the medium level of creativity, consequently the low level decreased. Some research linked the ability of people to experience atypical body expressions allowing them to explore new ideas without restrictions, thus enhancing creativity [41].

By segregating the sample by gender, a considerable initial difference between groups in terms of creativity was found. This fact might be related to the artistic, sporting preferences of women, which despite using repetitive or pre-established elements have a great creative content [16,42]. However, in studies carried out decades ago, it was considered that women lacked creative values, due to the low presence of women in the field of research, art, etc. [43]. Social and cultural pressures favored the creativity and performance of men and have hindered the creativity of women [44]. Even so, psychologists have observed that the genres were differentiated by the creative factors analyzed. Thus, women obtained a better score in environmental sensitivity, own strength, intellectuality and individuality [42], while men stood out in initiative and mastery [45].

After the body expression activity, male participants’ performance on the creativity measures generally was better than females, with significant differences [46,47]. The progress is even more remarkable if we mention that they started from an uneven base, with a lower evaluation. This fact could be related to the novelty that the subject represented for most men because they prefer activities with dominance of force and physical contact [13].

One study affirms that this difference was related to the level of testosterone of the cerebral development that can influence in the decision of the dominant cerebral hemisphere. However, this hypothesis has received little support in the community [12,48]. It must be mentioned that favorable evolutions were observed in the two groups. The progress made by the female subjects, despite being objectively remarkable, failed to obtain a degree of significance.

The differences between the practitioners of team sports and those of individual sports in the first evaluation were basically non-existent. Creativity has been shown as a common factor in the sports field, at the same time it has presented the great diversity of facets required depending on the specific characteristics of the sport [21]. While imagination, novelty and expressiveness represent the degree of creativity in artistic sports, in collective sports, the fluidity or speed of responses, surprise factor and variability are more relevant [49]. On the other hand, in the final evaluation, group sports practitioners stood out for their higher score than individual sports practitioners. Learning new movements that were participants were not used to do made them progress more in the field of creativity.

Although differences have been shown between genders and sports practice, the growth in scores has been generalized and, therefore, the method it is to be proposed to promote the practice of body expression at any other age levels [17].

## 5. Conclusions

Finally, the participants after intervention had shown progression of creativity in college age while practicing body expression. The results obtained have shown a greater capacity for creative growth on the part of men, since before attending body expression classes’ women had higher scores. It was also observed that in the final evaluation, team sports practitioners stood out for their higher score than individual sports practitioners.

## Figures and Tables

**Table 1:** CREA (creative intelligence test) scale for Spanish samples and corresponding levels of creativity [26].

**Direct Score of CREA A + CREA B**	**Percentile**	**Creativity Level**
65	99	High
60–64	98	
56–59	97	
54–55	96	
53	95	
46–52	90	
42–45	85	
40–41	80	
38–39	75	
36–37	70	Medium
34–35	65	
33	60	
32	55	
30	50	
29	45	
28	40	
26–27	35	
24–25	30	
23	25	Low
20–22	20	
19	15	
17–18	10	
15–16	5	
13–14	4	
12	3	
11	2	
0–10	1	

**Table 2:** The content of body expression classes.

1st Session	First Contact with the Subject with Disinhibition Activities Through Specific Games with High Component of Fun (Impressions, Mime)
2nd Session	Body knowledge and posture control: specific barre workout, stretching and body consciousness through relaxation techniques
3th Session	Discover the space and the ways of displacement: formations and levels of movement
4th Session	Learn to differentiate and follow different rhythms and intensities
5th Session	Relationships development within the choreography: equals, mirror, different, alike, and complementary
6th Session	Basic techniques used in traditional dances; coordination and creativity
7th Session	Representation of group choreography and evaluation

**Table 3:** General comparison between pre- and post-test of the creative intelligence levels.

Test		*n*	Mean	SD
**CREA Pre**	Low Level	25	17.40	4.406
	Medium Level	24	29.08	3.866
	High Level	0	-	-
**CREA Post**	Low Level	17	17.94	4.293
	Medium Level	30	30.04	4.081
	High Level	2	39	1.414

**Table 4:** Gender comparison between pre- and post-test of the creative intelligence levels.

Test	Level	Gender					
		Men			Women		
		*n*	Mean	SD	*n*	Mean	SD
CREA Pre	Low	18	17.39	4.368	7	17.43	4.860
	Medium	16	28.56	3.847	8	30.13	3.944
	High	0	-	-	0	-	-
CREA Post	Low	12	18	3.742	5	17.80	5.933
	Medium	21	30.05	4.189	9	30	4.065
	High	1	40	-	1	38	-

**Table 5:** Type of sport affiliation comparison between pre- and post-test CREA.

Sport	Test	*n*	Mean	SD	*p*	ES
Team	CREA Pre	29	22.93	6.41	0.001	0.6
	CREA Post	29	27.31	7.31		
Individual	CREA Pre	20	23.40	8.36	0.231	0.1
	CREA Post	20	24.60	7.69		

**Table 6:** Type of sport affiliation comparison between pre- and post-test of the creative intelligence levels.

Test		Sport					
		Team			Individual		
		*n*	MEAN	SD	*n*	MEAN	SD
CREA Pre	Low Level	15	18.13	4.274	10	16.30	4.596
	Medium Level	14	28.07	3.731	10	30.50	3.779
	High Level	0	-	-	0	-	-
CREA Post	Low Level	8	18.13	4.357	9	17.78	4.494
	Medium Level	19	29.95	3.965	11	30.18	4.468
	High Level	2	39	1.414	0	-	-

## Data Availability

The data are not publicly available because they are subject to the confidentiality law presented in the informed consent.
